# Supplementary material for: miR-26a Attenuated Bone-Specific Insulin Resistance and Bone Quality in Diabetic Mice
Source: Mol Ther Nucleic Acids. 2020 Mar 29;20:459–67. doi: 10.1016/j.omtn.2020.03.010 (PMC7150437; doi:10.1016/j.omtn.2020.03.010)
Supplement: Document S1. Figures S1 and S2 [file mmc1.pdf]

OMTN, Volume *20*

Supplemental Information

miR-26a Attenuated Bone-Specific Insulin Resistance and Bone Quality in Diabetic Mice

Fusong Jiang, Yang Zong, Xin Ma, Chaolai Jiang, Haojie Shan, Yiwei Lin, Wenyang Xia, Fuli Yin, Nan Wang, Lihui Zhou, Zubin Zhou, and Xiaowei Yu

Supplementary Materials

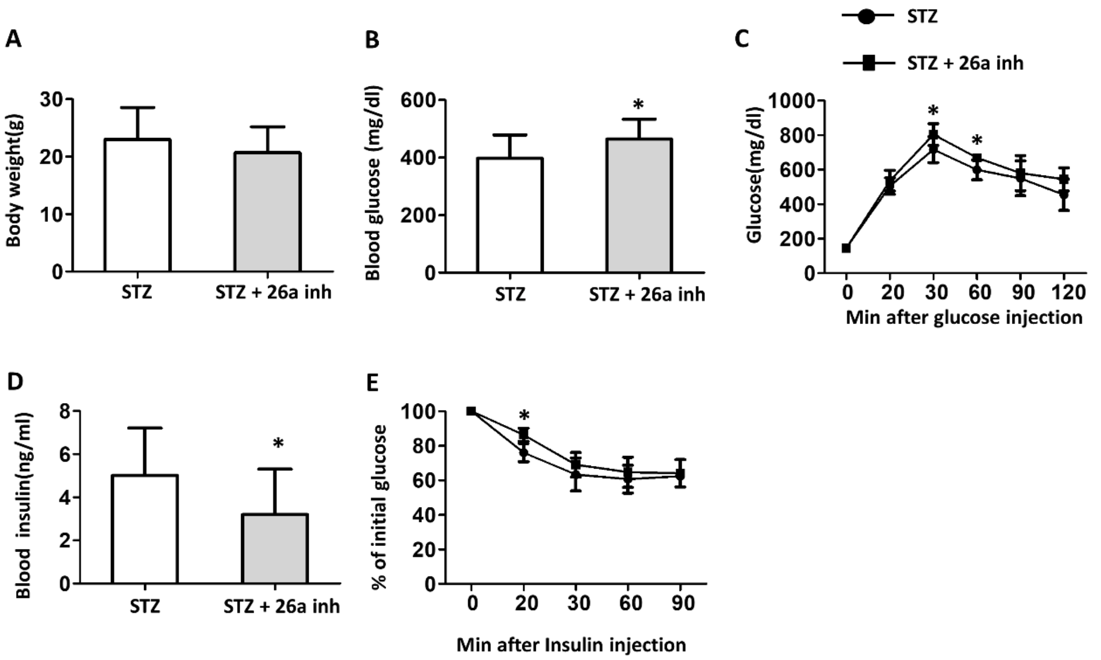

Figure S1. Effects of miR-26a inhibitor on insulin resistance and glucose tolerance in diabetic mice. (A) Total Body weight, (B) Blood glucose, (C) GTT, (D) Blood insulin and (E) ITT, performed after 18 weeks of STZ. Data are shown as mean ± SD. N=8 mice, *P < 0.05; **P < 0.01 compared with STZ group.

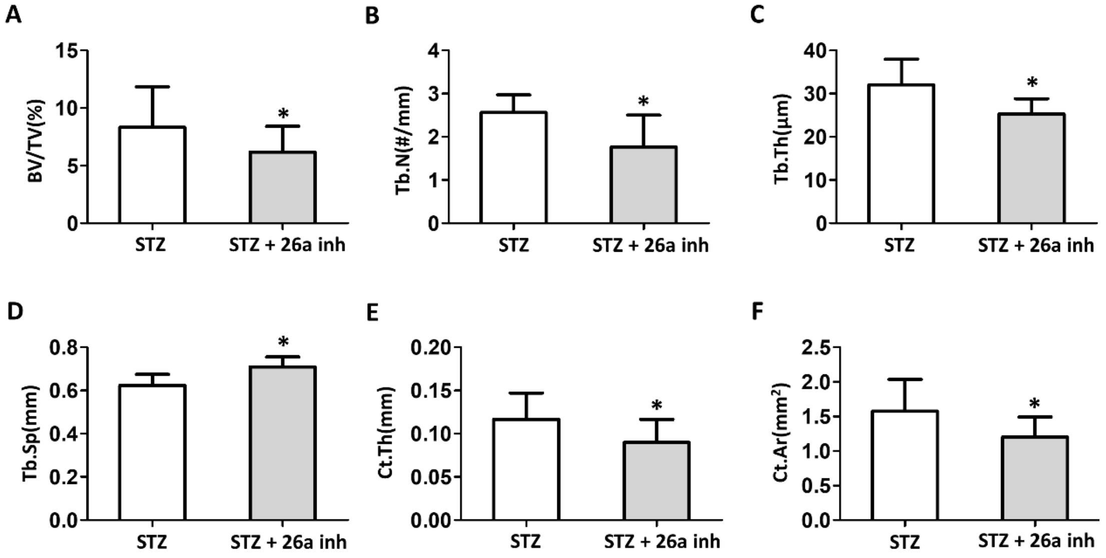

Figure S2. Effect of miR-26a inhibitor on trabecular bone microarchitecture in distal femora and cortical bone thickness of diabetic mice. (A) bone volume per tissue volume (BV/TV), (B) trabecular number (Tb.N), (C)trabecular thickness (Tb.Th), (D) trabecular separation (Tb.Sp), (E) cortical thickness (Ct.Th) and (F)cortical area (Ct.Th). Data are shown as mean ± SD. N=8 mice, *P < 0.05; **P < 0.01 compared with STZ group.
