# Supplementary material for: miR-26a Attenuated Bone-Specific Insulin Resistance and Bone Quality in Diabetic Mice
Source: Mol Ther Nucleic Acids. 2020 Mar 29;20:459–67. doi: 10.1016/j.omtn.2020.03.010 (PMC7150437; doi:10.1016/j.omtn.2020.03.010)
Supplement: Document S2. Article plus Supplemental InformationAuthor Contributions — F.J., Y.Z., X.M., C.J., H.S., Y.L., W.X., F.Y., N.W., L.Z., and Z.Z. performed the experiments and analyzed and interpreted the data. X.Y. was the major contributor in writing the manuscript. All authors read and approved the final manuscript. [file mmc2.pdf]

Molecular Therapy
Nucleic Acids
Original Article

miR-26a Attenuated Bone-Specific Insulin Resistance and Bone Quality in Diabetic Mice

Fusong Jiang,[1,5] Yang Zong,[2,5] Xin Ma,[2] Chaolai Jiang,[2] Haojie Shan,[2] Yiwei Lin,[2] Wenyang Xia,[2] Fuli Yin,[2] Nan Wang,[3] Lihui Zhou,[4] Zubin Zhou,[2] and Xiaowei Yu[2]

[1]Department of Endocrinology and Metabolism, Shanghai Jiao Tong University Affiliated Sixth People's Hospital, Shanghai Clinical Center for Diabetes, Shanghai 200233, China; [2]Department of Orthopaedic Surgery, Shanghai Jiao Tong University Affiliated Sixth People's Hospital, Shanghai 200233, China; [3]Department of Emergency, The First Affiliated Hospital of Zhengzhou University, Zhengzhou 450052, Henan, China; [4]Department of Orthopaedic Surgery, Xiangshan First People's Hospital, Ningbo 315700, Zhejiang, China

Diabetes mellitus is a prevalent disease result in several complications, including bone problems. Previous studies have shown that microRNA (miR)-26a regulates glucose metabolism and plays a protective role in diabetes. However, whether miR-26a also affects bone quality in diabetes remains unknown. In the present study, we evaluated the potential effects of miR-26a on bone in diabetic mice. We administrated miR-26a in streptozotocin-induced diabetic mice. The metabolic parameters, bone quality, osteoblast and osteoclast markers, and insulin signaling activation were measured. miR-26a ameliorated insulin resistance and glucose tolerance, improved bone microarchitecture and quality, increased osteoblasts and bone formation, decreased osteoclasts, and promoted the insulin signaling pathway in diabetic mice. These effects were abolished in insulin receptor-compromised Col1a1-Insr$^{+/-}$ mice. In conclusion, miR-26a could ameliorate bone-specific insulin resistance and bone quality in diabetic mice, which depended on the insulin receptors on osteoblasts. Our findings highlight the potential of miR-26a as a therapeutic target for diabetes mellitus-related bone metabolism and diseases.

INTRODUCTION

Diabetes mellitus (DM), which is commonly known as diabetes, is a metabolic disease characterized by high blood sugar.[1] DM is caused by the failure to produce enough insulin or the failure to respond to insulin properly. The prevalence of DM over the world is increasing, which has caused a great economic burden. Diabetes is also a major cause of cardiovascular disease, kidney failure, neuropathy, and blindness, which are significant causes of morbidity and mortality in diabetic patients.[2]

It has been also described that diabetes affects bone health. High serum glucose concentrations inhibit the production of bone-formation marker osteocalcin (OCN), which impaired bone formation.[3] Chronic hyperglycemia affects osteoblasts (OBs) maturation by downregulating osteocalcin expression.[4] Diabetes also affects bone microarchitecture by decreasing bone mineral density (BMD), which has been described in type 1 diabetes patients.[5] In a streptozotocin (STZ)-induced diabetic mice model, the growth factors, including insulin-like growth factor 1 (IGF-1) and transforming growth factor β1 (TGF-β1), are differentially expressed, which contributes to decreased bone growth.[6]

MicroRNA has been shown to regulate diabetes. The miR-26 family, which is composed of miR-26a and miR-26b, plays a crucial role in tumorigenesis by targeting critical regulators involved in development, cell cycle, and differentiation. In particular, miR-26a acts as a potent tumor suppressor in the liver, the central organ involved in maintaining glucose and lipid homeostasis.[7] miR-26a-overexpressing/transgenic mice have improved insulin sensitivity, decreased glucose level, and decreased fatty acid synthesis. In contrast, inhibition of miR-26a results in impaired insulin sensitivity and increased glucose level.[7]

miR-26a has been reported to participate in bone metabolism. For instance, miR-26a promotes osteogenic differentiation of unrestricted somatic stem cells isolated from human cord blood.[8] miR-26a could enhance bone regeneration in a mouse osteoporotic model.[9] Moreover, miR-26a mimics could promote osteogenic differentiation in nonunion rats.[10] These findings suggested the potential links between miR-26 and bone metabolism. However, the precise effects of miR-26a on the bone under diabetic conditions remain unknown. In the present study, the effects of miR-26a on bone metabolism in diabetic mice were evaluated.

RESULTS

miR-26a Ameliorated Insulin Resistance and Glucose Tolerance in Diabetic Mice

First, we evaluated the effects of miR-26a in STZ-induced diabetic mice. As shown in Figure 1A, STZ-treated mice had lower body

Received 17 March 2020; accepted 20 March 2020;
https://doi.org/10.1016/j.omtn.2020.03.010.

[5]These authors contributed equally to this work.

Correspondence: Xiaowei Yu, Department of Orthopaedic Surgery, Shanghai Jiao Tong University Affiliated Sixth People's Hospital, Shanghai 200233, China.
E-mail: yuxw@sjtu.edu.cn
Correspondence: Zubin Zhou, Department of Orthopaedic Surgery, Shanghai Jiao Tong University Affiliated Sixth People's Hospital, Shanghai 200233, China.
E-mail: zhouzubin@sjtu.edu.cn

This is an open access article under the CC BY license (http://creativecommons.org/licenses/by/4.0/).

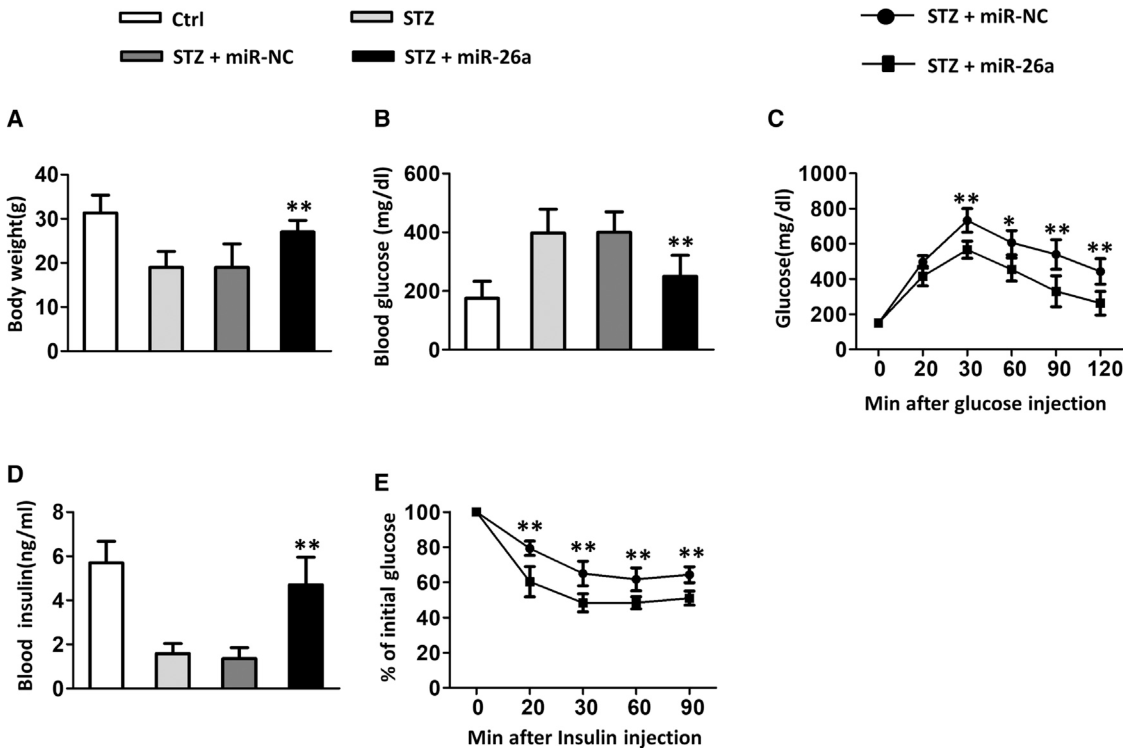

Figure 1. miR-26a Mimics Prevent Insulin Resistance and Glucose Tolerance in Diabetic Mice

(A) Total body weight, (B) blood glucose, (C) GTT, (D) blood insulin, and (E) ITT, performed after 18 weeks of STZ. Data are shown as mean ± SD. n = 8 mice, *p < 0.05; **p < 0.01 compared with STZ group.

weight compared to control mice. Administration of control microRNA (miRNA) did not affect the body weight of diabetic mice. In contrast, the administration of miR-26a by tail-vein injection resulted in significantly increased body weight in diabetic mice. Diabetic mice had a higher blood glucose level (Figure 1B), whereas the administration of miR-26a significantly decreased blood glucose levels. In contrast, diabetic mice had a lower blood insulin level (Figure 1D), whereas administration of miR-26a significantly increased blood insulin level. Furthermore, with the use of a glucose tolerance test (GTT) and insulin tolerance test (ITT), diabetic mice administrated with miR-26a had significantly improved glucose tolerance (Figure 1C) and insulin sensitivity (Figure 1E) when compared to control of diabetic mice.

To confirm further the effect of miR-26a in STZ-induced diabetic mice, we treated the mice with the miR-26a inhibitor (26a inh). The miR-26a inhibitor showed no influence on diabetic mice body weight (Figure S1A), whereas it significantly increased blood glucose concentration (Figure S1B). The miR-26a inhibitor worsened glucose tolerance (Figure S1C) and insulin sensitivity (Figure S1E) in the early stages of GTT and ITT assays, whereas no difference was observed in the late stages (Figures S1C and S1E). Moreover, miR-26a significantly suppressed the blood insulin levels in diabetic mice (Figure S1D). Taken together, our data demonstrated that miR-26a ameliorated insulin resistance and glucose tolerance in diabetic mice.

miR-26a Improved Trabecular Bone Microarchitecture in Distal Femora and Cortical Bone Thickness in Diabetic Mice

Next, we evaluated the effects of miR-26a on bone microarchitecture and bone thickness. Compared to normal mice, diabetic mice had significantly decreased bone volume per tissue volume (BV/TV) (Figure 2A), trabecular number (Tb.N) (Figure 2B), trabecular thickness (Tb.Th) (Figure 2C), and increased trabecular separation (Tb.Sp) (Figure 2D). Furthermore, diabetic mice had significantly lower cortical bone parameter cortical thickness (Ct.Th) (Figure 2E) and cortical area (Ct.Ar) (Figure 2F) when compared to normal mice. The administration of control miRNA did not affect these parameters in diabetic mice. In contrast, miRNA-26a significantly improved bone microarchitecture in diabetic mice by increasing BV/TV (Figure 2A), Tb.N (Figure 2B), and Tb.Th (Figure 2C) and decreasing Tb.Sp (Figure 2D). In addition, the administration of miRNA-26a significantly increased Ct.Th (Figure 2E) and Ct.Ar (Figure 2F) in diabetic mice.

Again, we explored the effect of 26a inh on diabetic mice. As expected, the miR-26a inhibitor showed the totally opposite effect to miR-26a administration, which is evidenced by decreased BV/TV (Figure S2A), decreased Tb.N (Figure S2B), decreased Tb.Th (Figure S2C), increased Tb.Sp (Figure S2D), decreased cortical bone parameters Ct.Th (Figure S2E), and decreased Ct.Ar (Figure S2F). Collectively, our data demonstrated that miR-26a improved bone microarchitecture bone thickness in diabetic mice.

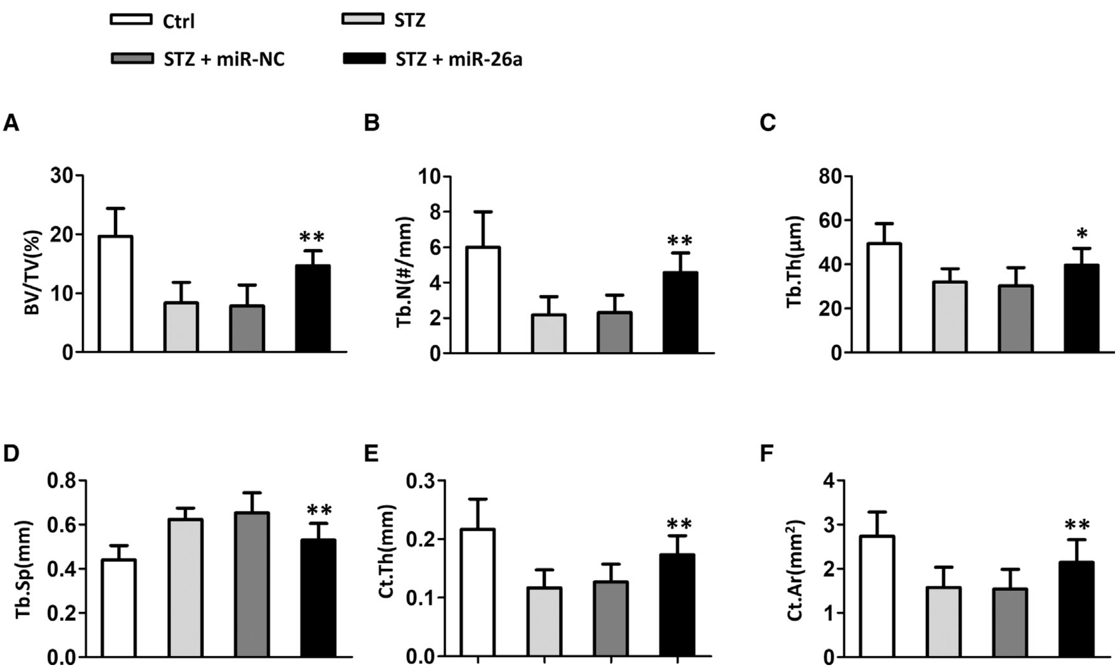

Figure 2. Effect of miR-26a Mimics on Trabecular Bone Microarchitecture in Distal Femora and Cortical Bone Thickness of Diabetic Mice

(A) Bone volume per tissue volume (BV/TV), (B) trabecular number (Tb.N), (C) trabecular thickness (Tb.Th), (D) trabecular separation (Tb.Sp), (E) cortical thickness (Ct.Th), and (F) cortical area (Ct.Th). Data are shown as mean ± SD. n = 8 mice, *p < 0.05; **p < 0.01 compared with STZ group.

miR-26a Increased Osteoblasts and Bone Formation and Decreased Osteoclasts in Diabetic Mice

We continued to evaluate how miR-26a affected bone microarchitecture by conducting the bone histomorphometry. In diabetic mice, the osteoblasts per bone surface (Nob/Bpm) (Figure 3A), osteoblast surface per bone surface (Obs/Bs) (Figure 3B), and osteoid surface per bone surface (OS/BS) (Figure 3C) were remarkably decreased. In contrast, the osteoclasts per bone surface (Noc/BS) (Figure 3D), osteoclast surface per bone surface (Ocs/BS) (Figure 3E), and eroded surface per bone surface (ES/BS) (Figure 3F) in diabetic mice were remarkably increased. Administration of miR-26a significantly increased the Nob/Bpm, Obs/Bs, and OS/BS, whereas it decreased the Noc/BS, Ocs/BS, and ES/BS in diabetic mice. Correspondingly, the diabetic mice had a significantly decreased mineralizing surface (MS/BS) (Figure 3G), mineral apposition rate (MAR) (Figure 3H), and bone formation rate (BFR) (Figure 3I), whereas administration of miR-26a significantly increased these parameters. Therefore, miR-26a increased osteoblasts and bone formation, whereas it decreased osteoclasts in diabetic mice.

miR-26a Affected Bone Resorption and Formation Marker Expression in Diabetic Mice

We continued to evaluate the effects of miR-26a on bone resorption and formation marker expression. Diabetic mice had remarkably increased the C-telopeptide fragments of type I collagen (CTX) level (Figure 4A) and decreased the OCN level (Figure 4B) and osterix level (Figure 4C) when compared to normal mice. miR-26a significantly decreased the CTX level and increased the OCN and osterix levels. Diabetic mice had remarkably decreased mRNA levels of osteoblast marker osteoprotegerin (OPG) (Figure 4D) and receptor activator of nuclear factor κB ligand (RANKL) (Figure 4E), whereas it had a remarkably increased the level of osteoclast marker tartrate-resistant acid phosphatase (TRAP) (Figure 4F) and Wnt antagonist sclerostin (Sost) (Figure 4G) and dickkopf-related protein 1 (Dkk1) (Figure 4H). In contrast, the OPG and RANKL mRNA levels were significantly increased in miR-26a-treated diabetic mice, whereas the mRNA levels of TRAP, Sost, and Dkk1 were significantly decreased in miR-26a-treated diabetic mice.

miR-26a Affected the Insulin Signaling Pathway in Diabetic Mice

Next, we evaluated whether miR-26a affected the insulin signaling pathway in bones. We injected insulin through the tail vein and tested the activation of the insulin signaling pathway. As shown in Figure 5A, injection of insulin in normal mice resulted in obviously increased phosphorylation of the insulin receptor and protein kinase B (AKT). In contrast, diabetic mice had less phosphorylation of the insulin receptor and AKT than normal mice after insulin injection. Administration of miR-26a in diabetic mice resulted in increased phosphorylation of both insulin receptor and AKT, indicating that miR-26a promoted an insulin signaling pathway. In addition, diabetic mice had less of an amount of total insulin receptors compared to normal mice, whereas miR-26a increased the total insulin receptor level. After quantitation, we detected that miR-26a significantly enhanced the phosphorylation of the insulin receptor (Figure 5B), total insulin receptor level (Figure 5C), and phosphorylation of AKT (Figure 5D).

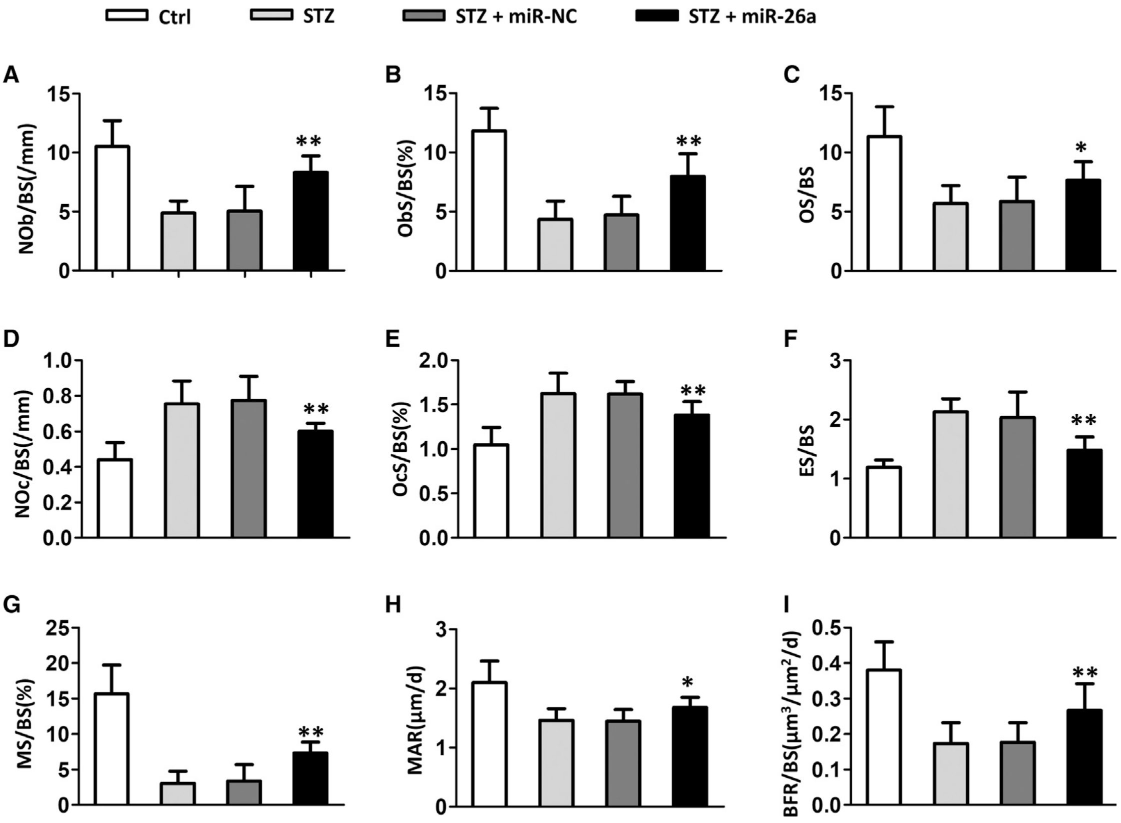

Figure 3. miR-26a Mimics Increased Osteoblasts and Bone Formation and Decreased Osteoclasts in Diabetic Mice

(A) Number of osteoblasts per bone surface (NOb/BS), (B) surface covered by osteoblasts (ObS/BS), (C) osteoid surface (OS/BS), (D) osteoclast number per bone surface (NOc/BS), (E) surface covered by osteoclasts (OcS/BS), (F) eroded surface per bone surface (ES/BS), (G) mineralizing surface (MS/BS), (H) mineral apposition rate (MAR), and (I) bone formation rate (BFR/BS). Data are shown as mean ± SD. n = 8 mice, $^{*}p < 0.05$; $^{**}p < 0.01$ compared with STZ group.

Compromising Insulin Signaling in Osteoblasts Abolished the Effects of miR-26a on Insulin Resistance and Glucose Tolerance in Diabetic Mice

As miR-26a affected the insulin signaling pathway in bones, we next asked whether the effects of miR-26a on insulin resistance and glucose tolerance depended on the insulin signaling pathway in osteoblasts. Therefore, mice lacking one allele of *Insr* in osteoblasts (Col1a1-Insr$^{+/-}$ mice) were used.[7] As shown in Figure 6, miR-26a significantly increased body weight (Figure 6A) and decreased glucose level (Figure 6B) in diabetic control Insr$^{fl/+}$ mice. In contrast, diabetic Col1a1-Insr$^{+/-}$ mice had higher body weight and glucose levels when compared to diabetic control Insr$^{fl/+}$ mice. In addition, the administration of miR-26a affected neither body weight nor blood glucose level of diabetic Col1a1-Insr$^{+/-}$ mice. With the use of the GTT, miR-26a-treated diabetic control Insr$^{fl/+}$ mice had better glucose tolerance than untreated diabetic control Insr$^{fl/+}$ mice (Figure 6C). Diabetic Col1a1-Insr$^{+/-}$ mice had worse glucose tolerance than diabetic control Insr$^{fl/+}$ mice, and miR-26a treatment did not affect glucose tolerance in diabetic Col1a1-Insr$^{+/-}$ mice. Similarly, miR-26a decreased the blood insulin level in diabetic control Insr$^{fl/+}$ mice, whereas it did not affect the blood insulin level in diabetic Col1a1-Insr$^{+/-}$ mice (Figure 6D). In the ITT, miR-26a improved insulin sensitivity in diabetic control Insr$^{fl/+}$ mice. Diabetic Col1a1-Insr$^{+/-}$ mice had worse insulin sensitivity than diabetic control Insr$^{fl/+}$ mice, and miR-26a treatment did not affect insulin sensitivity in diabetic Col1a1-Insr$^{+/-}$ mice (Figure 6E).

Compromising Insulin Signaling in Osteoblasts Abolished the Effects of miR-26a on Bone Microarchitecture and Bone Thickness in Diabetic Mice

We continued to evaluate whether the effects of miR-26a on bone health depended on the insulin signaling pathway in osteoblasts. miR-26a significantly increased the BV/TV (Figure 7A), Tb.N (Figure 7B), Tb.Th (Figure 7C), Ct.Th (Figure 7E), and Ct.Ar (Figure 7F), whereas it significantly decreased the Tb.Sp (Figure 7D) in diabetic control Insr$^{fl/+}$ mice. Diabetic Col1a1-Insr$^{+/-}$ mice have less BV/TV, Tb.N, Tb.Th, Ct.Th, and Ct.Ar, whereas they had more Tb.Sp than diabetic control Insr$^{fl/+}$ mice. Moreover, miR-26a did not affect BV/TV, Tb.N, Tb.Th, Ct.Th, Tb.Sp, and Ct.Ar in diabetic Col1a1-Insr$^{+/-}$ mice. Taken together, our data demonstrated that miR-26a affected bone microarchitecture and bone thickness depending on insulin signaling in osteoblasts.

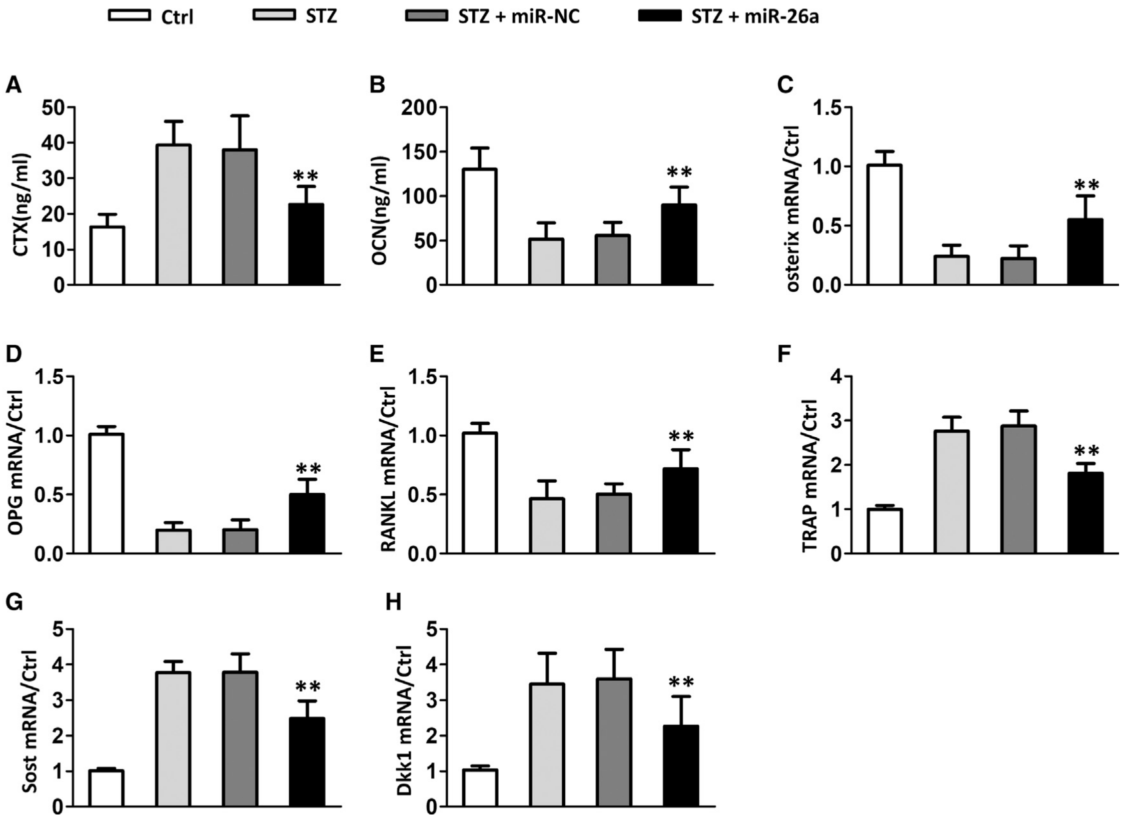

Figure 4. Effect of miR-26a Mimics on Bone Resorption and Formation Markers and Bone Gene Expression Induced by STZ

(A) C-telopeptide fragments of type I collagen (CTX) and (B) osteocalcin (OCN) measured at the end of the study in serum and plasma, respectively. (C–H) Expression of the osteoblast marker osterix (C) and the cytokines OPG (D) and RANKL (E), the osteoclast marker TRAP (F), and the Wnt/β-catenin antagonists, Sost (G) and Dkk1 (H), in tibia. Data are shown as mean ± SD. n = 8 mice, **p < 0.01 compared with STZ group.

DISCUSSION

DM is a prevalent disease causing a significant economic burden. There are also several comorbidities associated with DM, including stroke, artery disease, and neuropathy. In addition, diabetes also affects bone health regarding bone strength, bone turnover, bone mineral density, and bone structure. Prevention of these disabling complications has attracted more and more attention. In the current study, we evaluated the protective effects of miR-26a, a microRNA that has been shown to protect against diabetes, on bone health in a STZ-induced diabetic animal. We demonstrated that miR-26a not only ameliorated glucose and insulin levels but also improved bone microarchitecture and bone thickness in diabetic mice. miR-26a promoted osteoblasts and bone formation, whereas it prevented osteoclast differentiation. Furthermore, we demonstrated that miR-26a enhanced the insulin signaling pathway in bones. The insulin pathway in the osteoblast plays an essential role in miR-26a-mediated protection in diabetes and bone health, as a compromise of insulin signaling in osteoblasts abolished these effects.

OBs play essential roles in bone formation, including synthesizing collagen, mineralizing osteoid, and participating in bone remodeling. The effects of diabetes on OBs have been extensively described. In STZ-induced diabetic mice, the Wnt/β-catenin pathway, which is essential for osteoblast differentiation and bone formation, was downregulated.[11] The downregulation of the Wnt/β-catenin pathway was partially caused by the upregulation of the Wnt signaling inhibitor Sost and Dkk1.[11] We also identified the upregulated expression of Sost and Dkk1 in diabetic mice. The miR-26a prevented the upregulation of Sost and Dkk1, suggesting that miR-26a promoted osteoblast differentiation and bone formation.

It has been reported that serum levels of CTX, a marker of bone resorption, and osteocalcin, a marker of bone formation, were lower in patients with diabetes when compared to healthy people, indicating impaired bone turnover in patients with diabetes.[12] In the current study, we also identified the lower levels of CTX and osteocalcin in diabetic mice. miR-26a treatment increased the serum levels of both CTX and osteocalcin in diabetic mice, indicating that miR-26a promoted bone turnover. RANKL binds to RANK in preosteoclasts and promotes osteoclast differentiation.[13,14] OPG, a negative regulator of osteoclastogenesis, has been shown to be downregulated in STZ-induced diabetes in mice. In contrast, RANKL mRNA levels

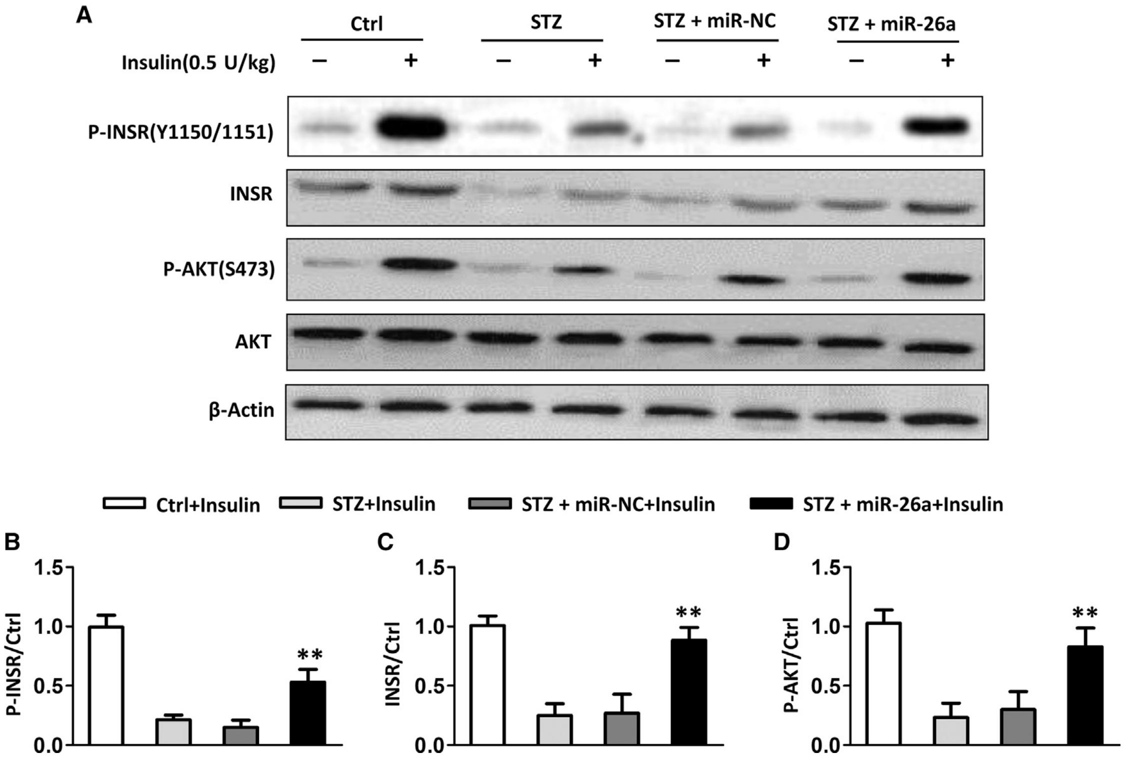

Figure 5. Effect of miR-26a Mimics on Insulin Signaling Induced by STZ

(A) Western blot analysis of the phosphorylation levels of INSR and AKT in bones. (B–D) Relative protein levels compared with the control (Ctrl) group. Data are shown as mean ± SD. n = 8 mice, **p < 0.01 compared with STZ group.

are increased in studies of STZ-induced diabetes. The dysregulated expression of these factors was ameliorated by miR-26a. miR-26a significantly increased the OPG but decreased RANKL expression in diabetic mice. Taken together, our findings demonstrated that miR-26a promoted osteoblast differentiation, inhibited osteoclast formation, and promoted bone formation, which contributed to enhanced bone health in diabetic mice.

The activities of miR-26a in the regulation of insulin sensitivity and glucose metabolism have been described.[7] Fu and colleagues[7] demonstrated that miR-26a directly targeted key regulators involved in insulin signaling, including protein kinase C (PKC)δ, PKCθ, and glycogen synthase kinase (GSK)3β. Insulin resistance condition activated PKCδ, PKCθ, and GSK3β, resulting in phosphorylation of insulin receptor substrate (IRS) proteins and attenuating insulin signaling.[15–17] miR-26a directly repressed PKCδ, PKCθ, and GSK3β expression, which contributed to the positive role of miR-26a in insulin signaling. The binding of insulin to its receptor triggered phosphorylation of the receptor and finally resulted in AKT phosphorylation.[18] Our current study also identified that miR-26a treatment promoted insulin signaling by increasing the phosphorylation of both insulin receptor and Akt. The insulin pathway played important roles in metabolic and mitogenic effects in many cell types, including osteoblasts. Insulin signaling regulated both bone formation by osteoblasts and bone resorption by osteoclasts, mainly through the insulin signaling pathway within osteoblasts.[19] Insulin signaling within an osteoblast has been shown to influence osteoblast proliferation, differentiation, and survival. Defective insulin signaling in osteoblast would result in impaired bone quantity and quality, as well as abnormal glucose metabolism. We also demonstrated that defective insulin signaling by compromising insulin receptors in the osteoblast also abolished the effects of miR-26a on the diabetic symptom and bone health in diabetic mice. Our results strongly suggested the essential role of the insulin signaling pathway within osteoblast in diabetes, indicating the osteoblast could function as a target for diabetes.

It is important to determine the limitations of this study. First, the present study only focuses on the effect of miR-26a on the indicated signaling pathways, whereas whether some other signaling pathways would be affected by miR-26a is still unknown. Second, the targeting genes of miR-26a were still not examined, and whether miR-26a has any off-target effects is also unknown. Third, the effect of miR-26a on diabetes should be verified using clinical samples.

Conclusions

miR-26a ameliorated insulin resistance and enhanced bone quality, which depended on insulin receptor/signaling in osteoblast.

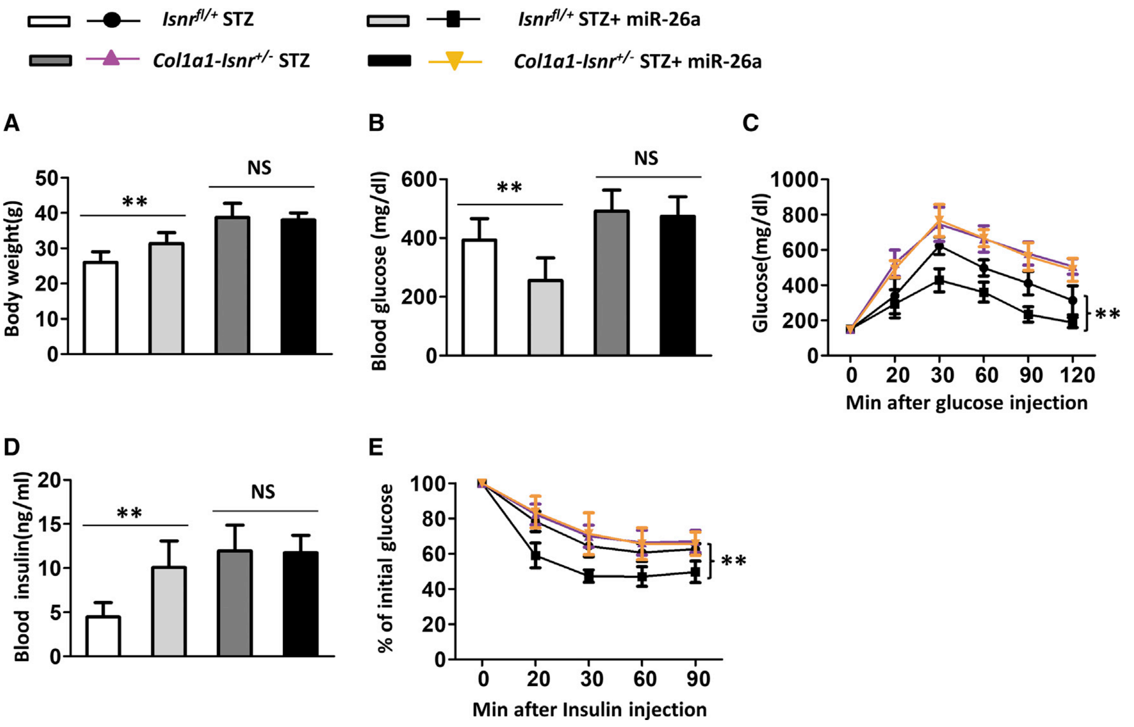

Figure 6. Effect of miR-26a Mimics on Insulin Resistance and Glucose Tolerance Was Blocked in Col1a1-Insr$^{+/-}$ STZ Mice

(A) Total body weight, (B) blood glucose, (C) GTT, (D) blood insulin, and (E) ITT performed after 18 weeks of STZ. Data are shown as mean ± SD. n = 8 mice. **p < 0.01. NS, no significance.

MATERIALS AND METHODS

Mice Treatment

Male C57BL/6 mice, 12 weeks of age, were purchased from Charles River (Beijing, China) and received a standard rodent diet and water al libitum. To induce type I diabetes, mice were injected STZ (45 mg/kg) in 50 mM citrate buffer (pH 4.5) daily for 5 days, as described previously.[20] Mice injected with citrate buffer alone were used as control. 18 days post-first STZ injection, the blood glucose concentration was measured to confirm the induction of diabetes using a portable glucose meter (Abbot Laboratories, Alameda, CA, USA). Mice with a nonfasting blood glucose level higher than 250 mg/dL were used as diabetic mice. *Col1a1-Insr*$^{+/-}$ mice were generated as described previously.[21] All animal studies were approved by the Ethical Committee in Shanghai Jiao Tong University Affiliated Sixth People's Hospital. In some experiments, mice were injected with control miRNA or miR-26a mimics (0.1 mg/kg, once a day, for 8 weeks) via the tail vein.

Metabolic Measurements

Ultra Sensitive Mouse Insulin ELISA Kit (Crystal Chem, Elk Grove Village, IL, USA) was used to measure the serum insulin levels. Blood glucose levels were measured using a portable glucose meter (Abbot Laboratories, USA). In the glucose tolerance test, after fasting for 16 h, mice were injected intraperitoneally with 2 g/kg D-glucose. For the insulin tolerance test, mice were fasted for 6 h and then injected intraperitoneally with 1 U/kg human insulin (Humulin R; Eli Lilly, Indianapolis, IN, USA).

Bone Histology and Histomorphometry

The vertebrae site of L3 and L4 was used for bone histology and histomorphometry analysis as described previously.[22] The Osteomeasure System (OsteoMetrics, Atlanta, GA, USA) and ImageJ were used to perform histomorphometry. Mineralized bone volume over the total tissue volume, osteoclast surface per bone surface, and bone formation rate per bone surface were measured as described previously.[23]

Microcomputed Tomography (μCT) Analysis

The trabecular and cortical bone architecture of proximal femurs was assessed using a μCT system (VivaCT 40; SCANCO Medical AG). Trabecular bone volume, cortical bone volume, and midshaft thickness were analyzed using the standard software provided by the manufacturer of the μCT scanner.

Western Blot

Total proteins from bones were isolated using Minute Total Protein Extraction Kit for Bone Tissue (Invent Biotech, Beijing, China) and then loaded on an SDS-PAGE gel. After transfer to a polyvinylidene fluoride (PVDF) membrane, the membranes were blocked in 5% non-fat milk at room temperature for 1 h. The primary antibodies,

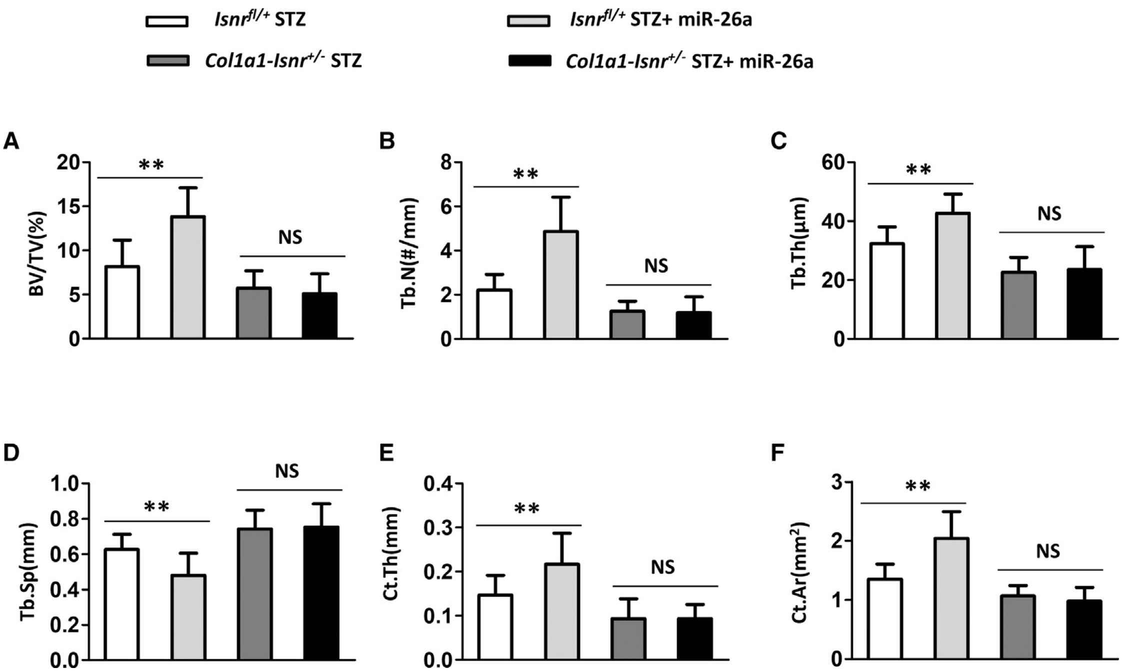

Figure 7. Effect of miR-26a Mimics on Trabecular Bone Microarchitecture in Distal Femora and Cortical Bone Thickness Was Blocked in Col1a1-Insr$^{+/-}$ STZ Mice

(A) Bone volume per tissue volume (BV/TV), (B) trabecular number (Tb.N), (C) trabecular thickness (Tb.Th), (D) trabecular separation (Tb.Sp), (E) cortical thickness (Ct.Th), and (F) cortical area (Ct.Ar). Data are shown as mean ± SD. n = 8 mice. **p < 0.01. NS, no significance.

including anti-phospho-insulin receptor antibody (Sigma, St. Louis, MO, USA), anti-insulin receptor antibody (Sigma, USA), anti-phospho-Akt antibody (Sigma, USA), anti-Akt antibody (Sigma, USA), and anti-β-actin (Sigma, USA), were added to the membranes for overnight at 4°C. The next day, membranes were washed with 0.1% Tris-buffered saline-Tween 20 (TBST), 3 times, and then incubated with corresponding horseradish peroxidase (HRP)-conjugated secondary antibodies for 1 h at room temperature. Immunoreactive proteins were detected by using the ECL (enhanced chemiluminescence) Western Blotting Substrate Kit (Abcam, Cambridge, MA, USA). The density was quantitated using ImageJ.

RT-PCR

The RNeasy Mini Kit (QIAGEN, Germantown, MD, USA) was used to extract total RNA from bones following the manufacturer's instructions. Then a reverse transcription kit (Takara, Tokyo, Japan) was used to reverse transcribe the isolated RNA. The QuantiTect SYBR Green RT-PCR Kit (QIAGEN, USA) was used to perform real-time PCR on a QuantStudio 3 Real-Time PCR System (Thermo Fisher Scientific, USA). Glyceraldehyde 3-phosphate dehydrogenase (GAPDH) was used as the internal control for relative expression normalization. The following primers for RT-PCR were purchased from GeneCopoeia (RiboBio, Guangzhou, China): RANKL: 5′-CACACCTCACCATCAATGCTGC-3′ (forward), 5′-GAAGGGTTGGACACCTGAATGC-3′ (reverse); OPG: 5′-TGCTCCTGGCACCTACCTAA-3′ (forward), 5′-TCACCTGAGAAGAACCCATCC-3′ (reverse); Sost: 5′-TGCCGCGAGCTGCACTACAC-3′ (forward), 5′-CACCACTTCACGCGCCCGAT-3′ (reverse); TRAP: 5′-TCCTGGCTCAAAAAGCAGTT-3′ (forward), 5′-ACATAGCCCACACCGTTCTC-3′ (reverse); Dkk1: 5′-TCACACCAAAGGACAAGAAGG-3′ (forward), 5′-CTTGGACCAGAAGTGTCTTGC-3′ (reverse); osterix: 5′-ATGGCGTCCTCTCTGCTTG-3′ (forward), 5′-TGAAAGGTCAGCGTATGGCTT-3′ (reverse); GAPDH: 5′-AGGTCGGTGTGAACGGATTTG-3′(forward), 5′-TGTAGACCATGTAGTTGAGGTCA-3′ (reverse).

ELISA

The CTX-I Assay Kit (Chondrex, Redmond, WA, USA) was used to measure the serum level of CTX. The Serum level of OCN was measured by using the Mouse Osteocalcin ELISA Kit (Novus Biologicals, Centennial, CO, USA).

Statistical Analysis

All data were presented as mean ± SD. Data were analyzed by one- or two-way ANOVA analysis, followed with a Bonferroni post hoc test. Statistical difference was considered significant when $p < 0.05$.

SUPPLEMENTAL INFORMATION

Supplemental Information can be found online at https://doi.org/10.1016/j.omtn.2020.03.010.

CONFLICTS OF INTEREST

The authors declare no competing interests.

ACKNOWLEDGMENTS

This work was funded by the Municipal Human Resources Development Program for Outstanding Leaders in Medical Disciplines in Shanghai (2018BR38), Foundation of Shanghai Health and Family Planning Commission (201740190), and National Natural Science Foundation of China (81873993).

REFERENCES

1. Yan, W., and Li, X. (2013). Impact of diabetes and its treatments on skeletal diseases. Front. Med. *7*, 81–90.
2. Deshpande, A.D., Harris-Hayes, M., and Schootman, M. (2008). Epidemiology of diabetes and diabetes-related complications. Phys. Ther. *88*, 1254–1264.
3. Inaba, M., Terada, M., Koyama, H., Yoshida, O., Ishimura, E., Kawagishi, T., Okuno, Y., Nishizawa, Y., Otani, S., and Morii, H. (1995). Influence of high glucose on 1,25-dihydroxyvitamin D3-induced effect on human osteoblast-like MG-63 cells. J. Bone Miner. Res. *10*, 1050–1056.
4. Botolin, S., and McCabe, L.R. (2006). Chronic hyperglycemia modulates osteoblast gene expression through osmotic and non-osmotic pathways. J. Cell. Biochem. *99*, 411–424.
5. Vestergaard, P. (2007). Discrepancies in bone mineral density and fracture risk in patients with type 1 and type 2 diabetes–a meta-analysis. Osteoporos. Int. *18*, 427–444.
6. Lu, H., Kraut, D., Gerstenfeld, L.C., and Graves, D.T. (2003). Diabetes interferes with the bone formation by affecting the expression of transcription factors that regulate osteoblast differentiation. Endocrinology *144*, 346–352.
7. Fu, X., Dong, B., Tian, Y., Lefebvre, P., Meng, Z., Wang, X., Pattou, F., Han, W., Wang, X., Lou, F., et al. (2015). MicroRNA-26a regulates insulin sensitivity and metabolism of glucose and lipids. J. Clin. Invest. *125*, 2497–2509.
8. Trompeter, H.I., Dreesen, J., Hermann, E., Iwaniuk, K.M., Hafner, M., Renwick, N., Tuschl, T., and Wernet, P. (2013). MicroRNAs miR-26a, miR-26b, and miR-29b accelerate osteogenic differentiation of unrestricted somatic stem cells from human cord blood. BMC Genomics *14*, 111.
9. Li, Y., Fan, L., Hu, J., Zhang, L., Liao, L., Liu, S., Wu, D., Yang, P., Shen, L., Chen, J., and Jin, Y. (2015). MiR-26a Rescues Bone Regeneration Deficiency of Mesenchymal Stem Cells Derived From Osteoporotic Mice. Mol. Ther. *23*, 1349–1357.
10. Sun, L., Li, Z., Xue, H., Ma, T., Ren, C., Li, M., Lu, Y., Sun, H., and Zhang, K. (2019). MiR-26a promotes fracture healing of nonunion rats possibly by targeting SOSTDC1 and further activating Wnt/β-catenin signaling pathway. Mol. Cell. Biochem. *460*, 165–173.
11. Hie, M., Iitsuka, N., Otsuka, T., and Tsukamoto, I. (2011). Insulin-dependent diabetes mellitus decreases osteoblastogenesis associated with the inhibition of Wnt signaling through increased expression of Sost and Dkk1 and inhibition of Akt activation. Int. J. Mol. Med. *28*, 455–462.
12. Hygum, K., Starup-Linde, J., Harsløf, T., Vestergaard, P., and Langdahl, B.L. (2017). MECHANISMS IN ENDOCRINOLOGY: Diabetes mellitus, a state of low bone turnover - a systematic review and meta-analysis. Eur. J. Endocrinol. *176*, R137–R157.
13. Pandolfi, F., Pitocco, D., Cianci, R., Mancini, L., and Ghirlanda, G. (2010). Increased osteoclastic activity in acute Charcot’s osteoarthopathy: the role of receptor activator of nuclear factor-kappa B ligand. Eur. Rev. Med. Pharmacol. Sci. *14*, 69–70.
14. Mabilleau, G., Petrova, N.L., Edmonds, M.E., and Sabokbar, A. (2008). Increased osteoclastic activity in acute Charcot’s osteoarthropathy: the role of receptor activator of nuclear factor-kappaB ligand. Diabetologia *51*, 1035–1040.
15. Johnson, A.M., and Olefsky, J.M. (2013). The origins and drivers of insulin resistance. Cell *152*, 673–684.
16. Guevara-Aguirre, J., and Rosenbloom, A.L. (2019). Branched Chain and Aromatic Amino Acids Are Associated With Insulin Resistance During Pubertal Development in Girls. J. Adolesc. Health *65*, 313–314.
17. Zhang, X., Ojanen, X., Zhuang, H., Wu, N., Cheng, S., and Wiklund, P. (2019). Branched-Chain and Aromatic Amino Acids Are Associated With Insulin Resistance During Pubertal Development in Girls. J. Adolesc. Health *65*, 337–343.
18. Nigi, L., Grieco, G.E., Ventriglia, G., Brusco, N., Mancarella, F., Formichi, C., Dotta, F., and Sebastiani, G. (2018). MicroRNAs as Regulators of Insulin Signaling: Research Updates and Potential Therapeutic Perspectives in Type 2 Diabetes. Int. J. Mol. Sci. *19*, E3705.
19. Pramojanee, S.N., Phimphilai, M., Chattipakorn, N., and Chattipakorn, S.C. (2014). Possible roles of insulin signaling in osteoblasts. Endocr. Res. *39*, 144–151.
20. Lozano, D., de Castro, L.F., Dapía, S., Andrade-Zapata, I., Manzarbeitia, F., Alvarez-Arroyo, M.V., Gómez-Barrena, E., and Esbrit, P. (2009). Role of parathyroid hormone-related protein in the decreased osteoblast function in diabetes-related osteopenia. Endocrinology *150*, 2027–2035.
21. Wei, J., Ferron, M., Clarke, C.J., Hannun, Y.A., Jiang, H., Blaner, W.S., and Karsenty, G. (2014). Bone-specific insulin resistance disrupts whole-body glucose homeostasis via decreased osteocalcin activation. J. Clin. Invest. *124*, 1–13.
22. Parfitt, A.M., Drezner, M.K., Glorieux, F.H., Kanis, J.A., Malluche, H., Meunier, P.J., Ott, S.M., and Recker, R.R. (1987). Bone histomorphometry: standardization of nomenclature, symbols, and units. Report of the ASBMR Histomorphometry Nomenclature Committee. J. Bone Miner. Res. *2*, 595–610.
23. Wei, J., Shi, Y., Zheng, L., Zhou, B., Inose, H., Wang, J., Guo, X.E., Grosschedl, R., and Karsenty, G. (2012). miR-34s inhibit osteoblast proliferation and differentiation in the mouse by targeting SATB2. J. Cell Biol. *197*, 509–521.

OMTN, Volume *20*

Supplemental Information

miR-26a Attenuated Bone-Specific Insulin Resistance and Bone Quality in Diabetic Mice

Fusong Jiang, Yang Zong, Xin Ma, Chaolai Jiang, Haojie Shan, Yiwei Lin, Wenyang Xia, Fuli Yin, Nan Wang, Lihui Zhou, Zubin Zhou, and Xiaowei Yu

Supplementary Materials

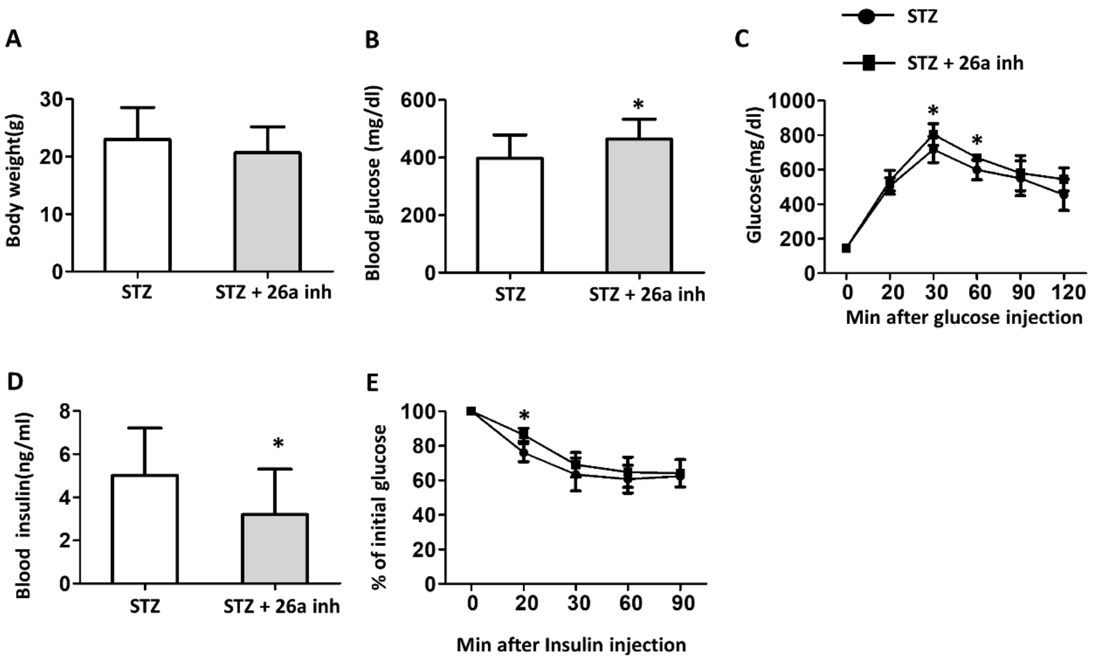

Figure S1. Effects of miR-26a inhibitor on insulin resistance and glucose tolerance in diabetic mice. (A) Total Body weight, (B) Blood glucose, (C) GTT, (D) Blood insulin and (E) ITT, performed after 18 weeks of STZ. Data are shown as mean ± SD. N=8 mice, *P < 0.05; **P < 0.01 compared with STZ group.

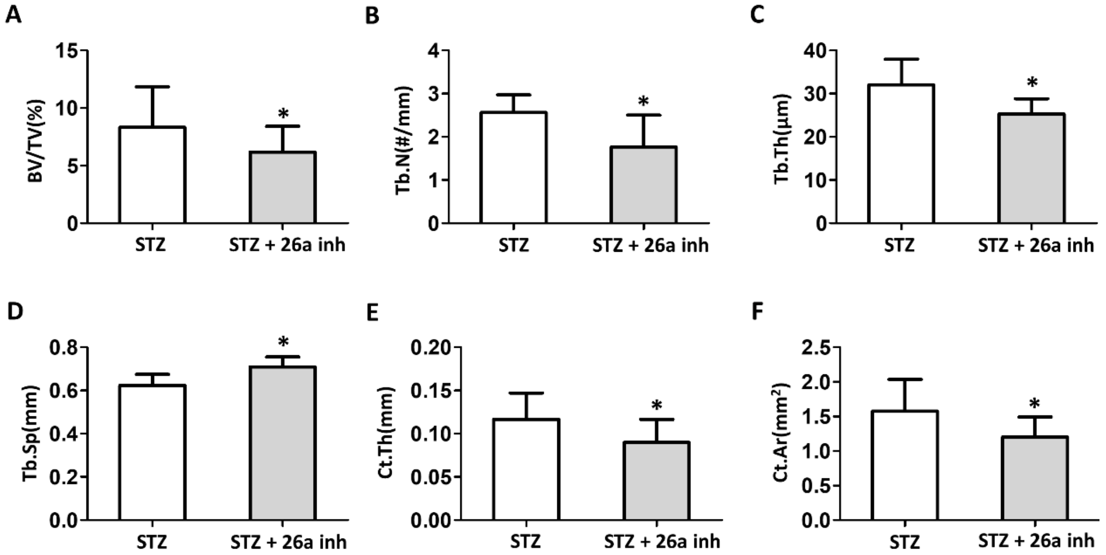

Figure S2. Effect of miR-26a inhibitor on trabecular bone microarchitecture in distal femora and cortical bone thickness of diabetic mice. (A) bone volume per tissue volume (BV/TV), (B) trabecular number (Tb.N), (C)trabecular thickness (Tb.Th), (D) trabecular separation (Tb.Sp), (E) cortical thickness (Ct.Th) and (F)cortical area (Ct.Th). Data are shown as mean ± SD. N=8 mice, *P < 0.05; **P < 0.01 compared with STZ group.
